# Phenotypic Diversity of 15q11.2 BP1–BP2 Deletion in Three Korean Families with Development Delay and/or Intellectual Disability: A Case Series and Literature Review

**DOI:** 10.3390/diagnostics11040722

**Published:** 2021-04-19

**Authors:** Ji Yoon Han, Joonhong Park

**Affiliations:** 1 Department of Pediatrics, College of Medicine, The Catholic University of Korea, Seoul 06591, Korea; han024@catholic.ac.kr; 2 Department of Laboratory Medicine, Jeonbuk National University Medical School and Hospital, Jeonju 54907, Korea; 3 Research Institute of Clinical Medicine of Jeonbuk National University-Biomedical Research Institute of Jeonbuk National University Hospital, Jeonju 54907, Korea

**Keywords:** 15q11.2 BP1–BP2 deletion, phenotypic diversity, development delay, intellectual disability, array comparative genomic hybridization

## Abstract

The 15q11.2 breakpoint (BP) 1–BP2 deletion syndrome is emerging as the most frequent pathogenic copy number variation in humans related to neurodevelopmental diseases, with changes in cognition, behavior, and brain morphology. Previous publications have reported that patients with 15q11.2 BP1–BP2 deletion showed intellectual disability (ID), speech impairment, developmental delay (DD), and/or behavioral problems. We describe three new cases, aged 3 or 6 years old and belonging to three unrelated Korean families, with a 350-kb 15q11.2 BP1–BP2 deletion of four highly conserved genes, namely, the *TUBGCP5*, *CYFIP1*, *NIPA2*, and *NIPA1* genes. All of our cases presented with global DD and/or ID, and the severity ranged from mild to severe, but common facial dysmorphism and congenital malformations in previous reports were not characteristic. The 15q11.2 BP1–BP2 deletion was inherited from an unaffected parent in all cases. Our three cases, together with previous findings from the literature review, confirm some of the features earlier reported to be associated with 15q11.2 BP1–BP2 deletion and help to further delineate the phenotype associated with 15q11.2 deletion. Identification of more cases with 15q11.2 BP1–BP2 deletion will allow us to obtain a better understanding of the clinical phenotypes. Further explanation of the functions of the genes within the 15q11.2 BP1–BP2 region is required to resolve the pathogenic effects on neurodevelopment.

## 1. Introduction

The proximal long arm of chromosome 15 is a region rich in segmental duplications that houses five breakpoints (BPs) for recurrent 15q copy number variations (CNVs) as defined by non-allelic homologous recombination. Microdeletions extending from BP1 to BP3 (type 1) or from imprinted region BP2 to BP3 (type 2) cause Prader–Willi syndrome/Angelman syndrome (PWS/AS), depending on the parental origin of the deleted allele [1]. The 15q13.3 microdeletion between BP4 and BP5, which constitutes the CHRNA7 gene, causes mild to moderate intellectual disability (ID) associated with epilepsy with various phenotypes [2]. The 15q11.2 BP1–BP2 deletion (Burnside–Butler) syndrome is emerging as the most frequent pathogenic CNV in humans related to neurodevelopmental diseases, with changes in cognition, behavior, and brain morphology [3]. Previous publications have reported that patients with the 15q11.2 BP1–BP2 deletion showed learning disabilities, speech impairment, developmental delay (DD), and/or behavioral problems [4,5,6,7]. Due to the various phenotypes, it is difficult to provide genetic counselling to families with the 15q11.2 BP1–BP2 deletion, particularly before prenatal diagnosis [8]. More than 200 15q11.2 deletion carriers have been described in clinical cases with mild, moderate, and severe neurodevelopmental manifestations as well as impairments, causing physicians to delineate a microdeletion syndrome with considerable variable expressivity [8] and incomplete penetrance [9]. Nevertheless, several patients carried another related genetic alteration and added confusion to genotype–phenotype correlations [7]. In addition, this CNV is frequently inherited from an unaffected parent; thus, the pathogenicity of the 15q11.2 BP1–BP2 deletion seems unclear, leading to its interpretation as a variant of uncertain significance.

In this report, we describe three new cases of 15q11.2 BP1–BP2 deletion of four highly conserved genes, namely, the *TUBGCP5*, *CYFIP1*, *NIPA2*, and *NIPA1* genes. All of our cases presented with global DD and/or ID, and the severity ranged from mild to severe, but the common facial dysmorphism and congenital malformations in previous reports were not characteristic.

## 2. Materials and Methods

### 2.1. Samples and DNA Extraction

We evaluated three unrelated cases diagnosed as DD/ID belonging to three different families referred to the Department of Pediatric Neurology, Daejeon St. Mary’s Hospital (Daejeon, Korea). Genomic DNA samples were obtained from leukocytes of peripheral blood using a QIAamp DNA Mini Kit (Qiagen GmbH, Hilden, Germany) according to the standard DNA isolation process. Their quantity and quality were estimated using a Qubit 2.0 Fluorometer with the Qubit dsDNA HS Assay kit and the TaqMan RNase P Detection Reagents kit (ThermoFisher Scientific, Waltham, MA, USA), and they were considered appropriate when the genomic DNA concentration was >10 ng/µL.

### 2.2. Array Comparative Genomic Hybridization

To identify the potential genetic cause of DD/ID in the three probands and their family members, array comparative genomic hybridization (CGH) was used to provide candidates for a first-tier clinical diagnostic test. We performed whole-genome screening of chromosomal rearrangements by array CGH using a SurePrint G3 Human CGH + SNP Microarray 4 × 180 K (Agilent Technologies, Santa Clara, CA, USA) according to the manufacturer’s instructions. All samples were matched with a human genomic DNA reference (Agilent Technologies or Promega, Madison, WI, USA). Data were obtained using the Agilent Feature Extraction software 12.0.2.2 and Agilent CytoGenomics 4.0 and visually assessed using the Agilent Genomic Workbench Software 7.0.4.0 and Agilent CytoGenomics 4.0. CNVs were identified using the ADM-2 algorithm, with filters of a minimal absolute average log ratio of 0.25 as a cut-off, >5 Mb of copy number neutral loss of heterozygosity regions, and a minimal size of 200 kb in the region. Genomic positions were mapped using the human genomic reference sequence GRCh37/hg19.

### 2.3. Exome Sequencing

In our three families, all of the probands were symptomatic, whereas their parents had no symptoms. If both parents are carriers of an autosomal recessive inheritance, this phenomenon could explain why they had an affected child. Thus, trio exome sequencing was performed to resolve this situation using the Agilent SureSelect XT Human All Exon kit v5 (Agilent Technologies, Santa Clara, CA, USA). Paired-end sequencing was conducted on the Illumina HiSeq 2500 system (Illumina, San Diego, CA, USA) to identify the genetic alteration, given the suspicion of neuropsychiatric disorder at the Green Cross Genome (Yongin, Korea). Base calling, alignment, variant calling, annotation, and quality control reporting were performed using a GATK Best Practices workflow for germline short variant discovery (https://gatk.broadinstitute.org/hc/en-us; accessed on 6 September 2020). The interpretation of sequence variants was manually reviewed by medical laboratory geneticists according to standards and guidelines from the Joint Consensus Recommendation of the American College of Medical Genetics and Genomics and the Association for Molecular Pathology [10]. Nucleotide changes that pass the filtering criteria are as follows: Phred quality score >20, no Fisher strand bias, read depth > 30×, allele frequency <0.1%, and non-synonymous substitution or indel occurred as compound heterozygous or homozygous state in coding region and exon–intron boundaries.

## 3. Case Presentation

We identified the 15q11.2 BP1–BP2 deletion in children from three unrelated Korean families, including two females and one male. We performed careful etiologic investigations of laboratory studies, and all parameters were within the normal range in all three cases. The laboratory studies included complete blood counts, electrolytes, thyroid function tests, lactate/pyruvate, arterial blood gases, plasma amino acid, urine organic acids, and carnitine profiles, and radiologic tests were conducted, including chest and spine X-rays. A length mutation analysis for *FMR1* (CGG)n triplet repeat status was conducted using AmplideX *FMR1* PCR reagents (Asuragen, Austin, TX, USA) and showed normal results (<45 repeats). Chromosomal analysis revealed a normal karyotype. The results of brain magnetic resonance imaging, auditory, and ophthalmological tests for our cases were normal.

**Figure 1:**
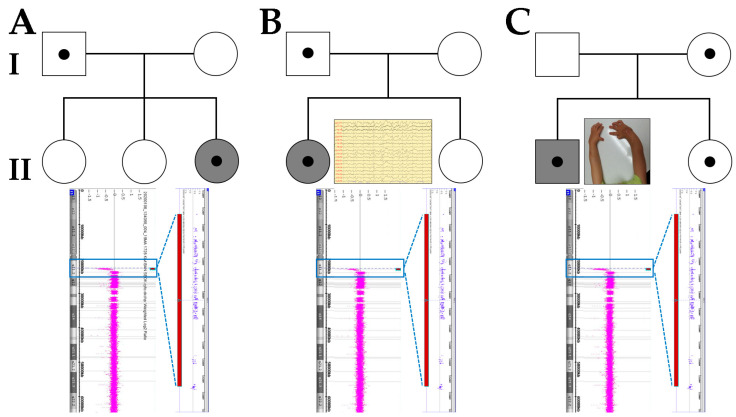
Family pedigree depicting 15q11.2 breakpoint (BP) 1 and BP2 deletion in three unrelated Korean families (**A**–**C**) with cases of developmental delay/intellectual disability. The grey symbol indicates an affected individual (upper panel). Array comparative genomic hybridization identified 15q11.2 BP1 and BP2 deletion in each proband (A-II-3, B-II-1, and C-II-1). The array 15q11.2q (22,784,523_23,179,948)×1 involving the genes *TUBGCP5*, *NIPA1*, *NIPA2*, and *CYFIP1* (lower panel).

Case A-II-3 (upper panel in Figure 1), a female diagnosed with DD at 3 years of age with DD, was referred for genetic diagnosis. She is a third child of non-consanguineous parents, and there is no family history of neurodevelopmental disorders. She was born via normal vaginal delivery at 39 weeks following an uneventful pregnancy. During infancy, she showed mild hypotonia and gross motor delay. She could control her head at 4 months, sit alone at 9 months, and walked at about 18 months. Her speech was delayed, and she could not speak in full sentences before she was 3 years old. She could speak only a few words at 40 months. At the age of 5 years, she had mild ID with an intelligence quotient (IQ) of 60 and attended an elementary school with additional specialized education at 7 years old. She had neither facial dysmorphism nor skeletal abnormalities. Her body weight was 34 kg (over 97th percentile), height was 119 cm (75th percentile), and head circumference was 52 cm (75th percentile). Her body mass index was 24 (over 97th percentile) and indicated obesity. The girl inherited the 15q11.2 BP1–BP2 deletion from her unaffected father. Her siblings have not been investigated.

Case B-II-1 (upper panel in Figure 1), a 6-year-old female with DD, was referred for genetic diagnosis. She is a first child of non-consanguineous parents, and there is no family history of neurodevelopmental disorders. The pregnancy was uneventful, with normal fetal movements. She was born via normal vaginal delivery at 40 weeks, and her birth weight was 3.5 kg. During infancy, early signs of developmental delay were noticed by the parents. She could roll over at 6 months and crawled at 14 months. She experienced unprovoked generalized tonic-clonic seizures at 6 months, and treatment with valproic acid was administrated for 1 year. She spoke a single word, “Mama”, at 22 months, and ten or more words at 30 months. She ran fairly well, stooped without bumping into things or falling at 30 months, and walked downstairs alone at 36 months. We examined her using the Bayley scale of infant and toddler development, third edition (Bayley-III), at the age of 48 months and found global DD (cognitive, motor, and language developmental ages: 13–18 months, 23–32 months, and 16–20 months, respectively). She formed sentences that were two words long at age 6. Formal psychometric tests at 6 years of age revealed an IQ of 45, and she attends a specialized primary school. She had no facial dysmorphism and showed a normal growth pattern. Her body weight was 20 kg (50th percentile), height was 112 cm (25th percentile), and head circumference was 52 cm (75th percentile). The girl inherited the 15q11.2 BP1–BP2 deletion from her unaffected father. Her sister does not have this deletion.

Case C-II-1 (upper panel in Figure 1), a 3-year-old male with DD and congenital agenesis of the radius noted at birth, was referred for genetic diagnosis. The pregnancy was uneventful, and his parents are both healthy and non-consanguineous. During pregnancy, there was no exposure to drugs, alcohol, or tobacco. He was born at 39 + 5 weeks via normal spontaneous delivery, and his birth weight was 2590 g. After the birth, he revealed congenital agenesis radius and hypoplastic carpal bones of both forearms. He exhibited torticollis, and a neck sonogram identified sternocleidomastoid muscle thickening. An echocardiogram showed a known cause of cardiac murmur and showed an atrial septal defect (type: ostium secundum). The atrial septal defect was closed 4 weeks after the birth. Special evaluation for skeletal dysplasia including laboratory tests and imaging was within the normal range. We examined him using the Bayley-III scale at the age of 39 months and found global DD (cognitive, motor, and language developmental ages: 19–22 months, 23–26 months, and 15–19 months, respectively). He had fine motor impairment in throwing a small ball, stacking small blocks, and making a mark on paper with a crayon at 42 months. His body weight was 13.5 kg (3rd percentile), height was 100 cm (10 to 25th percentile), and head circumference was 48.5 cm (10th percentile) at age 4. He attended additional education programs, including speech therapy, fine motor exercise, and social skill training. The boy inherited the 15q11.2 BP1–BP2 deletion from his unaffected mother. His sister (age of 32 months) also has this deletion, but is phenotypically unaffected (Table 1).

## 4. Results

Following the genetic analyses, the array CGH identified an approximately 350-kb microdeletion located between BP1 and BP2 in the PWS/AS critical region and involved the same four highly conserved genes, namely, *TUBGCP5*, *NIPA1*, *NIPA2,* and *CYFIP1*, in all three cases (lower panel in Figure 1). However, there were no likely shared pathogenic candidate variants identified by trio exome sequencing or correlations between genotype and clinical phenotype in any of the three cases with the 15q11.2 BP1–BP2 deletion.

## 5. Discussion

**Figure 2:**
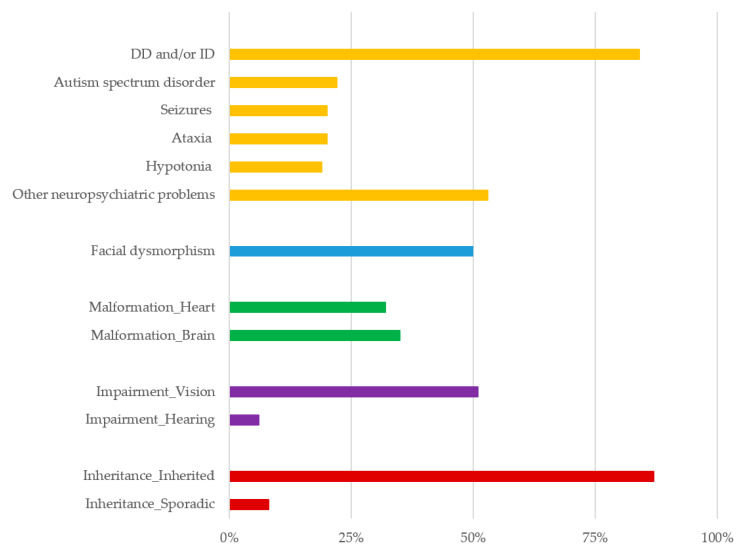
Literature review of the clinical characteristics and frequencies in 141 reported cases with 15q11.2 breakpoint (BP) 1 and BP2 deletion [4,5,6,7,12,13,14,15,16]. DD, developmental delay; ID, intellectual disability.

Various mechanisms affect the generation of CNVs, including non-allelic homologous recombination involving low copy DNA repeats, moving element insertions, non-homologous end connecting, folk stalling, and template switching [11]. The 15q11.2-q13 region is one of the genomic hotspots for CNVs. Literature review [4,5,6,7,12,13,14,15,16] of the clinical characteristics and frequencies in 141 reported cases with 15q11.2 breakpoint (BP) 1 and BP2 deletion is illustrated in Figure 2. Patients with a 15q11.2 BP1–BP2 deletion show very different clinical manifestations. Particularly, most cases with the 15q11.2 BP1–BP2 deletion are found at under 18 years of age and show some degree of DD/ID or neuropsychiatric problems. DD/ID were significantly more prevalent in a 15q11.2 BP1–BP2 deletion cohort than in a non-deleted control cohort [17]. Additionally, children with the 15q11.2 BP1–BP2 deletion showed behavioral problems, including attention deficit hyperactivity disorder (ADHD), autism spectrum disorder, obsessive compulsive disorder (OCD), and other psychiatric problems, at a proportion of 70% [18,19]. Seizures were noted in 20% of the patients and associated malformation of the brain was noted in 35%. Additionally, 15q11.2 BP1–BP2 deletion affects the brain structure; therefore, some patients have presented with psychosis or schizophrenia [12,20]. Dysmorphic features were reported in 30 to 50% of the patients and were quite varied, including dysmorphic ear, hypertelorism, high arched palate, and/or micrognathia [13,14]. However, a review of the literature indicated that 43% of these individuals had abnormal brain imaging such as magnetic resonance imaging and computed tomography and electroencephalography, with common clinical features of seizures or epilepsy (26%) and ataxia or coordination problems [8,15].

The clinical importance of a pure 15q11.2 BP1–BP2 deletion has been argued. Jønch, A.E., and colleagues recommended that the deletion should be classified as pathogenic of a mild effect size because it explains only a small proportion of the phenotypic variance in carriers [21]. On the contrary, 15q11.2 BP1–BP2 structural variation is associated with cognition and brain morphology, with deletion carriers being particularly affected. The pattern of results fits with the known molecular functions of genes in the 15q11.2 BP1–BP2 region and suggests a contribution of these genes to the association of this CNV with neurodevelopmental disorders [22]. In this report, our cases showed a pathological nature, although there was, apparently, an incomplete penetrance. These parents were completely normal from birth to adolescence and showed no learning difficulties, no facial dysmorphic features, nor any neurodevelopmental disabilities. However, all of our patients showed global DD and/or ID, but the severity ranged from mild to severe, and the common facial dysmorphism and congenital malformations in previous reports were not characteristic of our cases. All of the patients showed mild hypotonia and gross motor delay during infancy, after which, with age, motor delay reached the normal range at around 3 years of age. However, this phenomenon is not severe compared with PWS patients. One patient (B-II-1) diagnosed with infantile epilepsy was treated with anti-seizure medication. In epilepsy during infantile period, nearly 40% of cases are known to be due to genetic factors [23]. Chromosomal imbalances or gene mutations that are associated with ion channels, including *SCN1A*, *SCN2A*, *SCN8A*, *KCNT1*, *KCNQ2*, and *FOXG1*, could lead to seizures. Patients with the 15q11.2 BP1–BP2 deletion had uncommon non-neurological manifestations that included esophageal atresia, cataracts, tracheoesophageal fistula, and congenital arthrogryposis [16,24,25]. We described the first non-neurological presentation of congenital absence of the radius, which helps us understand more expanded features. Hypoplasia or aplasia of the radius were related to some genetic causes that participate in the early phases of skeletal patterning or upper limb growth [26]. About half of radial disorders have a Mendelian inheritance, whereas the remaining half occur sporadically, with no identified genes [27]. All patients with a Mendelian inheritance have syndromic forms, such as Holt–Oram syndrome, Fanconi anemia, platelet deficiency, and Okihiro syndrome, and these are associated with genetic causes such as trisomy 18, 13, *HOX*, *WNT*, and *TBX* [28]. The patient (C-II-1) with congenital agenesis of the radius could be interconnected with neurological dysfunction related to disturbed genes located on 15q11.2 BP1–BP2 and may be considered an extension of the clinical phenotypes. In this study, we performed trio exome sequencing to identify same candidate variants associated with DD/ID, dysplasia of radius, or epilepsy. However, no likely identical pathogenic variants were detected in all three cases, even though we attempted to explain the fact that the patients have different severities and despite having the same genetic cause. Several reports have highlighted the pathological nature of 15q11.2 deletion, although there is obviously an incomplete penetrance [5,8,9,29]. Additionally, we suggested that 15p11.2 can be a possible genetic cause of neurodevelopmental delay and other combined features.

The 15q11.2 BP1–BP2 region showed incomplete penetrance; low penetrance of pathogenicity has been estimated at 10.4% [9]. All of our cases were inherited from unaffected parents, and penetrance was low in each family. Several hypotheses exist for the incomplete penetrance and different expressivity noted in 15q11.2 BP1–BP2 deletion [5]. A parent or their offspring may be mildly affected and not explore medical consideration. Unequal parental expression of one or more genes can cause specific phenotypes. Incomplete penetrance and differences of expression require further assessment and research. Parent-of-origin effects of the 15q11.2 BP1–BP2 deletion are related to phenotypic differences in clinical features in individuals carrying this deletion. Sex-based differences were observed, such as macrocephaly, epilepsy, and autism spectrum disorder seen in maternal deletions and congenital heart disease, and abnormal muscular phenotypes seen in paternal deletions [29]. The inheritance pattern of our three families does not indicate suspicious genomic imprinting according to the sex of the parent and parent-specific expression. The 15q11.2 BP1–BP2 deletion included four genes, namely, *TUBGCP5*, *CYFIP1*, *NIPA1*, and *NIPA2*, and these genes are not related to imprinting mechanisms.

**Figure 3:**
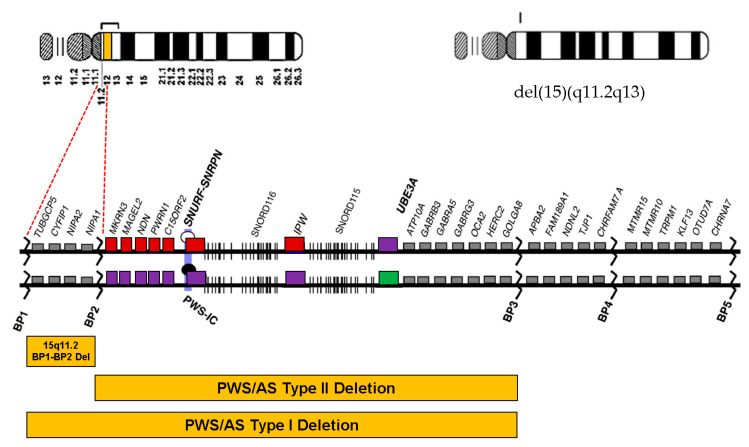
Map of the 15q11.2-q13 region demonstrates gene distribution between breakpoint (BP) 1 and BP5 on the proximal 15q11.2 region. The 15q11.2 deletion region is located between BP1 and BP2. Genes displayed in red and green boxes are imprinted and expressed from the paternal and maternal alleles, respectively. Violet boxes indicate the silenced alleles. Gray boxes mark genes expressed from both parental alleles. Del, deletion; AS, Angelman syndrome; BP, breakpoint; PWS, Prader–Willi syndrome; IC, imprinting center.

These four genes located on the 15q11.2 BP1–BP2 region are highly conserved (Figure 3). When these genes are disturbed, various neuropsychiatric problems seem to occur. Tubulin complex-associated protein 5 (*TUBGCP5*) encodes the gamma-tubulin complex component 5 that is part of the gamma-tubulin complex, which is necessary for microtubule nucleation at the centrosome [30,31]. *TUBGCP5* is dominantly expressed in nuclei of the sub-thalamic areas, which have been related to ADHD and OCD [32]. The cytoplasmic FMRP interacting protein 1 (*CYFIP1*) gene encodes a protein that regulates multiple actions in the cell, including organization of the actin cytoskeleton, maturation, and stabilization of dendritic spines [33,34]. *CYFIP1* has been shown to interact with ras-related C3 botulinum toxin substrate 1 (RAC1), which is expressed in the development and maintenance of dendritic fine structures. *CYFIP1/2* is present in synaptic extracts [33]. It can affect various neurodevelopmental pathways, and a large chromosomal deletion including this gene caused dysregulation that raised risk of schizophrenia and seizures in patients [35]. Non-imprinted in PWS/AS region 1 (*NIPA1*) encodes a magnesium transporter that confers with early endosomes and the cell surface in neurons and epithelial cells [36]. This protein may play a critical role in nervous system development and maintenance. Gain-of-function mutations of this gene have been associated with autosomal dominant spastic paraplegia 6 [37,38]. Two of the four genes (i.e., *NIPA1* and *NIPA2*) are expressed in the brain and encode magnesium transporters. Anecdotally, parents have administered magnesium supplements to their children with the 15q11.2 BP1–BP2 deletion and have observed improvements in behavior and clinical presentation [39]. Some reports identified an association pending confirmation about a variant in the *NIPA2* gene in patients with childhood absence seizures or generalized epilepsy [40,41]. Recently, in the results of in silico analyses of the functions and interactions of these four protein-coding genes in this region, all four genes were associated with up to three-fourths of ten overlapping neurodevelopmental disorders and were deleted in this most prevalently known pathogenic copy number variation, now recognized among humans with these clinical findings [3]. Recent studies introduced polygenic risk scores for neurodevelopmental disorders and explained the genetic correlation between DD/ID and autistic features [42,43]. Future studies delineating the phenotype associated with 15q11.2 deletion are needed to establish genetic liabilities of the 15q11.2 BP1–BP2 region for neurodevelopmental problems—for instance, quantitative PCR analysis or gene dosage tests such as multiplex ligation-dependent probe amplification of the deleted genes to understand possible differences that might justify the phenotype, or others. Our study had the limitation of a small number of cases; therefore, further phenotype studies with larger cohorts are required.

## 6. Conclusions

In conclusion, our three new cases, together with previous findings from the literature review, confirm some of the features earlier reported to be associated with 15q11.2 BP1–BP2 deletion and help to further delineate the phenotype associated with 15q11.2 deletion. Identification of more cases with the 15q11.2 BP1–BP2 deletion will allow us to obtain a better understanding of the clinical phenotypes. Further explanation of the functions of the genes within the 15q11.2 BP1–BP2 region is required to resolve the pathogenic effects on neurodevelopment.

## Figures and Tables

**Table 1:** Comparison of the clinical manifestations between three cases with 15q11.2 breakpoint (BP) 1 and BP2 deletion.

Clinical Manifestations	A-II-3	B-II-1	C-II-1
Sex/Age (Year) at Diagnosis	F/3	F/6	M/3
Inheritance	Paternal	Paternal	Maternal
Parent Status	Unaffected	Unaffected	Unaffected
Height < −2SD	Absent	Absent	Absent
Obesity	Present	Absent	Absent
Auxology			
IUGR	Absent	Absent	Absent
Failure to Thrive	Absent	Absent	Absent
Microcephaly	Absent	Absent	Absent
Facial Dysmorphism			
High Forehead	Absent	Absent	Absent
Hypertelorism	Absent	Absent	Absent
Dysplastic Ears	Absent	Absent	Absent
Long Philtrum	Absent	Absent	Absent
High Arched Palate	Absent	Absent	Absent
Micrognathia	Absent	Absent	Absent
Cleft Palate/lip	Absent	Absent	Absent
Deformity/Impairments			
Brain	Absent	Absent	Absent
Heart	Absent	Absent	Absent
Vision	Absent	Absent	Absent
Hearing	Absent	Absent	Absent
Neurology–Psychiatry			
Intellectual Disability	Present	Present	Present
Ataxia	Absent	Absent	Absent
Seizures	Absent	Present	Absent
ASD	Absent	Absent	Absent
ADHD	Absent	Absent	Absent
OCD	Absent	Absent	Absent
Other Psychobehavioral Problems	Absent	Absent	Absent
Neurodevelopment			
Hypotonia	Present	Present	Present
Delayed Motor Milestones	Present	Present	Present
Speech Impairment	Present	Present	Present
Learning Difficulties	Present	Present	Present

M, male; F, female; SD: Standard Deviation; IUGR: intrauterine growth retardation; ASD: autism spectrum disorder; ADHD: attention deficit hyperactivity disorder; OCD: obsessive compulsive disorder.

## Data Availability

The data presented in this study are available upon request from the corresponding author.
